# Supplementary material for: Use of generative AI for health among urban youth in Pakistan: A mixed-methods study
Source: PLOS Digit Health. 2026 Apr 6;5(4):e0001353. doi: 10.1371/journal.pdig.0001353 (PMC13052884; doi:10.1371/journal.pdig.0001353)
Supplement: S1 Text — (PDF) [file pdig.0001353.s002.pdf]

S1 Text. Full quantitative survey instrument and analysis variables.

Listed below are the questions asked in the quantitative survey tool of this research study, followed by a list of variables categorized by each section.

CONSENT

Consent: By clicking 'Yes,' I confirm that I am 18–30 years old and voluntarily agree to participate in this anonymous study on AI and health.

SECTION I: ABOUT YOU

Q1: What is your age?

Q2: What is your gender?

Q3: Which of the following best describes your sexual orientation?

Q4: Which of the following boards did you study from?

Q5: How many people in your life would you consider close friends or companions you trust?

Q6: Do you feel comfortable discussing sensitive or personal health concerns with your family?

Q7: Do you have any existing or past medical or mental health conditions (diagnosed or undiagnosed)?

Q8: Have you ever used any of the following non-generative AI or tech-enabled health tools? (Select all that apply)

Q9: How often do you use AI tools like ChatGPT, DeepSeek, Gemini, etc?

SECTION II: USE OF GENERATIVE AI FOR HEALTH

Q1: Have you ever used a generative AI platform to ask about a health-related concern?

Q2: Which AI platform(s) have you used for health-related questions? (Select all that apply.)

Q3: When you've used AI for health-related concerns, who was it for?

Q4: What types of health issues have you asked about using AI? (Select all that apply.)

Q5: What first led you to try an AI platform for a health-related concern? (Select all that apply)

Q6: How helpful did you find the AI's response to your health concern?

Q7: How frequently do you use AI for health-related concerns?

Q8: What do you usually do after receiving health-related advice from an AI platform?

Q9: Compared to visiting a doctor, how comfortable do you feel asking sensitive health questions to an AI platform?

SECTION III: TRUST IN AI

Q1: How much do you/would you trust the information provided by AI platforms like ChatGPT for health-related questions?

Q2: How confident are you about using AI platforms to find and understand health-related information?

Q3: Are you aware of any potential risks of using AI platforms, either in general or for health-related concerns?

Q4: Which of the following are you concerned about regarding usage of AI for health? (Select all that apply)

SECTION IV: ACCESS TO HEALTHCARE

Q1: If you needed to see a doctor today, how easy would it be for you to arrange that on your own?

Q2: Overall, how satisfied are you with the healthcare services available to you?

Q3: How often does getting professional healthcare for a health concern end up being delayed or avoided in your case?

Q3b: What are the most common reasons why you have delayed or avoided seeking healthcare? (Select the top 3 options)

LUCKY DRAW ENTRY

Optional: Would you like to enter a lucky draw for a small token of appreciation.

Section 1 variables: age, gender, sexual_orientation, education_board, num_close_friends, comfortable_sharing_health_with_family, existing_health_conditions, non_generative_health_tool_use, ai_tool_usage_frequency

Section 2 variables: used_ai_for_health, ai_platforms_used_for_health, who_used_ai_for, health_issues_asked_about, reason_for_using_ai_for_health, ai_response_helpfulness, frequency_of_ai_use_for_health, action_after_ai_advice, comfort_comparing_ai_vs_doctor

Section 3 variables: trust_in_ai_for_health, confidence_in_using_ai_for_health, aware_of_ai_health_risks, ai_health_concerns

Section 4 variables: ease_of_arranging_doctor_visit, satisfaction_with_healthcare_services, frequency_of_delaying_healthcare, reasons_for_delaying_healthcare
