# Supplementary material for: Use of generative AI for health among urban youth in Pakistan: A mixed-methods study
Source: PLOS Digit Health. 2026 Apr 6;5(4):e0001353. doi: 10.1371/journal.pdig.0001353 (PMC13052884; doi:10.1371/journal.pdig.0001353)
Supplement: S2 Fig — (PDF) [file pdig.0001353.s003.pdf]

S2 Fig. Good Reporting of a Mixed-Methods Study checklist.

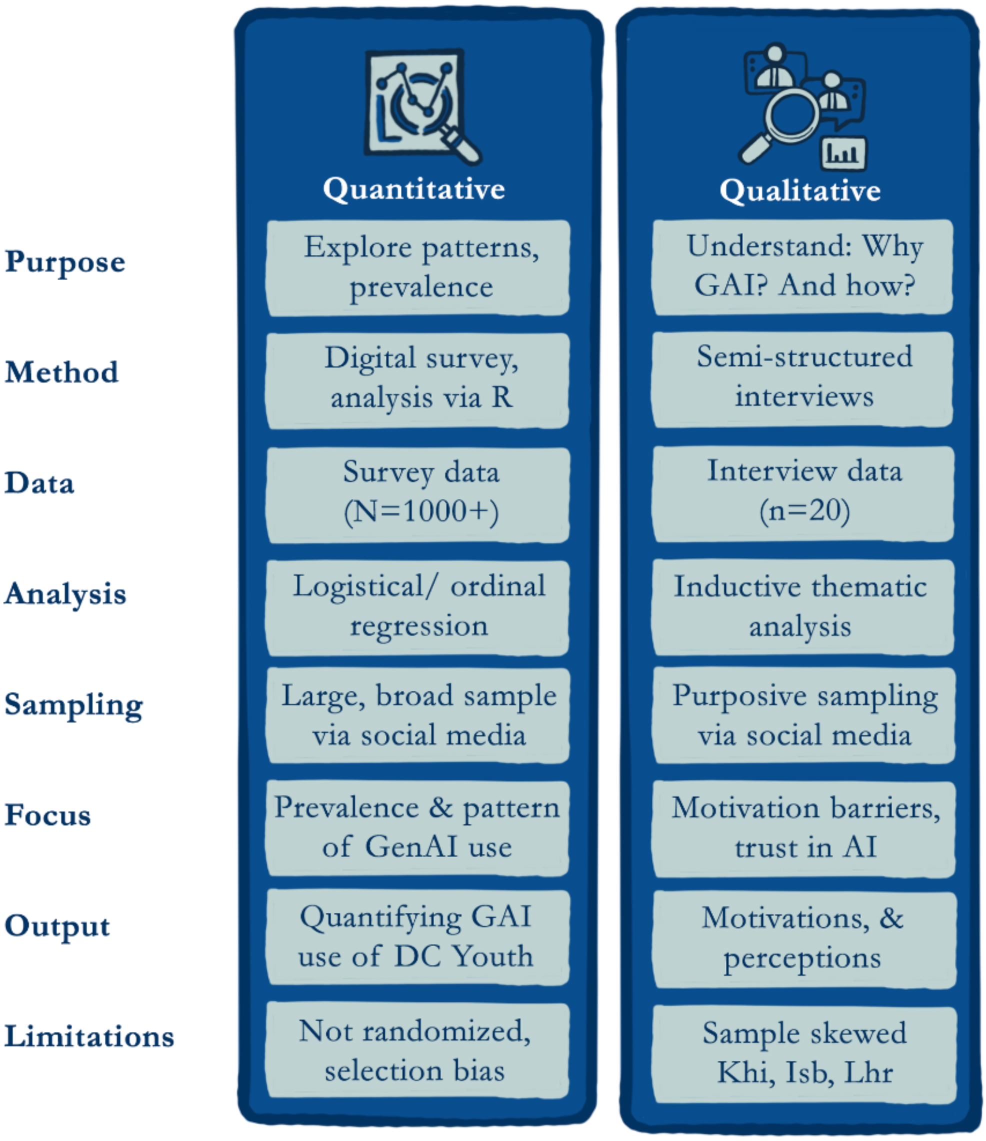

S2 Fig. Good Reporting of a Mixed-Methods Study checklist.
