# Supplementary material for: Use of generative AI for health among urban youth in Pakistan: A mixed-methods study
Source: PLOS Digit Health. 2026 Apr 6;5(4):e0001353. doi: 10.1371/journal.pdig.0001353 (PMC13052884; doi:10.1371/journal.pdig.0001353)
Supplement: S1 Table — (PDF) [file pdig.0001353.s004.pdf]

S1 Table. COREQ 32-item checklist.

Item #	COREQ item	Response for this study
1	Interviewer/facilitator	All five authors (AM, AA, IK, MH, SB) conducted interviews.
2	Credentials	Undergraduate/early-career researchers (ages 20–30) at Habib University; trained in qualitative methods and YPAR. AM (PRS DPhil), AA, IK, MH, SB (BSc Social Development Policy)
3	Occupation	Students/research fellows at Habib University during the study.
4	Gender of interviewer	Mixed team (women and men).
5	Experience/training	Team training in semi-structured interviewing, reflexive TA; piloting and cognitive testing conducted.
6	Relationship established	Minimal/No prior relationships with participants.
7	Participant knowledge of interviewer	Participants informed the study was youth-led, academic, non-profit; aims, roles, and confidentiality explained in consent.
8	Interviewer characteristics/reflexivity	Pakistani youth researchers; digitally connected; kept reflexive memos to bracket assumptions.
9	Methodological orientation	Reflexive Thematic Analysis (Braun & Clarke) with an inductive approach; socio-ecological model guided inquiry.
10	Sampling	Purposive/self-selection of urban youth (18–30) who reported routine GAI use for health.
11	Method of approach	Social media recruitment (stories/posts); scheduling via messages/email; virtual interviews (Trello).
12	Sample size	n = 20 interviews.

13	Non-participation	Not systematically recorded; some initial contacts did not schedule or later withdrew due to availability. No formal refusals documented.
14	Setting of data collection	Virtual (Zoom/Google Meet) to maximize geographic access and comfort.
15	Presence of non-participants	None.
16	Sample description	Urban young adults (18–30), gender-mixed; digitally connected; routine GAI users; pseudonyms assigned.
17	Interview guide	Semi-structured guide developed from formative work; piloted and refined. (Guide available on request; domains reported in Methods 2.2.)
18	Repeat interviews	None.
19	Audio/visual recording	Yes: audio-recorded with consent.
20	Field notes	Yes: taken during and after interviews.
21	Interview duration	~30–60 minutes.
22	Data saturation	Not used; adequacy judged via information power: later interviews added nuance, not new themes.
23	Transcript return	No transcript/member checking (to preserve anonymity and minimize burden).
24	Number of data coders	Team-based analysis; AA. IK MH SB. AM all coded.
25	Coding tree description	Inductive codebook iteratively developed and refined via team discussions and memoing; available on request.
26	Theme derivation	Inductive; themes developed through reflexive engagement with data.
27	Software	Mixed: Turboscribe for transcription; shared docs/spreadsheets and memoing for

		analysis (no dedicated CAQDAS); R used for quant only.
28	Participant checking of findings	No (see item 23 rationale).
29	Quotations presented	Yes—illustrative quotes with pseudonyms and basic descriptors included.
30	Data–finding consistency	Clear alignment between quotes, codes, and reported themes.
31	Clarity of major themes	Three major themes reported (Access/affordability; Emotional safety/support; Empowerment/agency).
32	Clarity of minor themes	Minor/divergent signals noted (e.g., climate anxiety; mixed signals on prior tool use).
