# Supplementary material for: Use of generative AI for health among urban youth in Pakistan: A mixed-methods study
Source: PLOS Digit Health. 2026 Apr 6;5(4):e0001353. doi: 10.1371/journal.pdig.0001353 (PMC13052884; doi:10.1371/journal.pdig.0001353)
Supplement: S2 Text — (PDF) [file pdig.0001353.s005.pdf]

S2 Text. Additional quantitative analyses and diagnostics.

Trust in AI was measured on a three-point Likert scale ("Not at all", "Somewhat", "A lot") and coded as an ordinal predictor (trust_num, 0–2) in the primary model. To evaluate the linearity assumption on the logit scale, we compared the pre-specified model (with trust as a 0–2 ordinal term) to an otherwise identical model in which "trust in AI" was entered as a three-level categorical factor. The categorical specification provided a better fit (ΔDeviance = 12.64 on 1 df, p = 0.00038; AIC = 1168.0 for the ordinal model vs 1157.4 for the categorical model). Nonetheless, the direction and magnitude of the trust effect were consistent with a strong monotonic gradient, and the adjusted odds ratios for all other predictors were very similar between models (ratios of adjusted odds ratios ranged from 0.99 to 1.16).

Model performance predictive metrics are summarised below:

- Discrimination (AUC): The area under the ROC curve was 0.740, indicating moderate discrimination between youth who did and did not use generative AI for health.
- Overall accuracy (Brier score): The Brier score was 0.177, suggesting reasonable overall accuracy of the predicted probabilities.

We explored two theory-driven interaction terms in the multivariable logistic regression:

1. Gender × any health condition (gender_simple × any_condition), and
2. Trust in AI × confidence using AI (trust_num × confidence_bin).

We fitted an extended model including both interactions and compared it to the pre-specified main-effects model using a likelihood ratio test.

The main-effects model had a residual deviance of 1136.0 on 1056 degrees of freedom, whereas the interaction model had a residual deviance of 1132.2 on 1054 degrees of freedom.

The likelihood ratio test indicated, as expected, no statistically significant improvement in fit (ΔDeviance = 3.86 on 2 df, p = 0.145). The number of parameters increased from 15 to 17, reducing the events-per-parameter ratio from 21.9 to approximately 19.3, while the main-effect adjusted odds ratios remained very similar between models.

Overall, these results provide no strong evidence that the examined interactions meaningfully improve the model, and we therefore retain the pre-specified main-effects model as primary in the main manuscript. The total quantitative sample comprised 1,240 participants aged 18–30 years. All descriptive statistics in the main manuscript are based on this full eligible sample. For the multivariable logistic regression, we used a complete-case analysis restricted to participants who (i) identified their gender as "Man" or "Woman" and (ii) had non-missing data on all variables in the pre-specified main-effects model. This yielded an analysis sample of N = 1,072, of whom 744 (69.4%) had used generative AI for health and 328 (30.6%) had not.

The final main-effects model includes 15 parameters (excluding the intercept), resulting in an events-per-parameter (EPP) ratio of 21.9, calculated as the number of events in the smaller outcome group (328 non-events) divided by the number of parameters.

This EPP comfortably exceeds standard rules-of-thumb for logistic regression. The table below shows the missingness for all variables if they had any missingness. As expected in the Pakistani context, sexual orientation had the highest missingness. Our formative research showed similarly i.e., individuals were most hesitant disclosing sexuality, even if online.
