# Supplementary material for: Use of generative AI for health among urban youth in Pakistan: A mixed-methods study
Source: PLOS Digit Health. 2026 Apr 6;5(4):e0001353. doi: 10.1371/journal.pdig.0001353 (PMC13052884; doi:10.1371/journal.pdig.0001353)
Supplement: S2 Table — (PDF) [file pdig.0001353.s006.pdf]

S2 Table. Trust in AI sensitivity analyses.

Predictor	OR_ord	p_ord_f	OR_cat	p_cat_f	OR_ratio_fmt
**Woman (vs Man)**	1.57 [1.17, 2.11]	0.003	1.55 [1.15, 2.09]	0.004	0.99
**LGBTQ+ (vs Heterosexual)**	0.59 [0.34, 1.05]	0.068	0.62 [0.35, 1.11]	0.100	1.04
**Other (vs Heterosexual)**	2.61 [0.35, 53.47]	0.410	2.77 [0.35, 58.63]	0.392	1.06
**International board (vs Local)**	1.12 [0.83, 1.51]	0.455	1.11 [0.82, 1.51]	0.493	0.99
**Other board (vs Local)**	0.40 [0.07, 1.80]	0.245	0.46 [0.09, 2.05]	0.320	1.16
**High social support (vs Low)**	0.91 [0.65, 1.29]	0.601	0.94 [0.67, 1.34]	0.740	1.03
**Any condition (Yes vs No)**	1.82 [1.34, 2.48]	<0.001	1.89 [1.39, 2.59]	<0.001	1.04
**Delay: Sometimes (vs Often)**	0.92 [0.63, 1.34]	0.678	0.95 [0.65, 1.39]	0.795	1.03
**Delay: Rarely (vs Often)**	0.91 [0.62, 1.35]	0.647	0.91 [0.61, 1.35]	0.635	1.00
**Delay: Never (vs Often)**	0.62 [0.35, 1.10]	0.102	0.66 [0.37, 1.17]	0.151	1.06
**Confidence: High (vs Low)**	1.81 [1.11, 3.07]	0.022	2.43 [1.43, 4.28]	0.001	1.34
**Aware of AI risks (Yes vs No)**	1.67 [1.20, 2.31]	0.002	1.65 [1.19, 2.29]	0.003	0.99
**Telemedicine user (vs None)**	1.58 [1.01, 2.54]	0.049	1.57 [0.99, 2.52]	0.059	0.99
**Other tool user (vs None)**	4.48 [2.59, 8.23]	<0.001	4.57 [2.63, 8.48]	<0.001	1.02
