# Supplementary material for: Use of generative AI for health among urban youth in Pakistan: A mixed-methods study
Source: PLOS Digit Health. 2026 Apr 6;5(4):e0001353. doi: 10.1371/journal.pdig.0001353 (PMC13052884; doi:10.1371/journal.pdig.0001353)
Supplement: S3 Table — (PDF) [file pdig.0001353.s007.pdf]

S3 Table. Interaction models.

term	estimate	std.error	statistic	p.value	conf.low	conf.high
gender_simpleWoman:any_conditionYes	1.259866329605	0.3047053258524	0.7581279620724	0.44837438158544	0.69211072939094	2.2875707284618
trust_num:confidence_binHigh	0.4189408416090	0.4723944582448	-1.8417353195420	0.06551387519701	0.16552323128026	1.0702892499278

S3 Table. Interaction models.
