# Supplementary material for: Use of generative AI for health among urban youth in Pakistan: A mixed-methods study
Source: PLOS Digit Health. 2026 Apr 6;5(4):e0001353. doi: 10.1371/journal.pdig.0001353 (PMC13052884; doi:10.1371/journal.pdig.0001353)
Supplement: S4 Table — (PDF) [file pdig.0001353.s008.pdf]

S4 Table. Missing data by variable.

variable	pct_missing
Q3. Which of the following best describes your sexual orientation?	3.3716475095785400
Q3b. What are the most common reasons why you have delayed or avoided seeking healthcare? (Select the top 3 options)	2.9118773946360200
Q1. Have you ever used a generative AI platform to ask about a health-related concern?	2.2222222222222200
Q3. How often does getting professional healthcare for a health concern end up being delayed or avoided in your case?	1.3793103448275900
Q2. Overall, how satisfied are you with the healthcare services available to you?	0.996168582375479
Q8. Have you ever used any of the following non-generative AI or tech-enabled health tools? (Select all that apply)	0.9195402298850580
Q1. If you needed to see a doctor today, how easy would it be for you to arrange that on your own?	0.8429118773946360
Q3. Are you aware of any potential risks of using AI platforms, either in general or for health-related concerns?	0.7662835249042150
Q4. Which of the following are you concerned about regarding usage of AI for health? (Select all that apply)	0.7662835249042150
Q2. How confident are you about using AI platforms to find and understand health-related information?	0.5363984674329500
Q7. Do you have any existing or past medical or mental health conditions (diagnosed or undiagnosed)?	0.38314176245210700
Q6. Do you feel comfortable discussing sensitive or personal health concerns with your family?	0.30651340996168600
Q1. How much do you/would you trust the information provided by AI platforms like ChatGPT for health-related questions?	0.30651340996168600
Q5. How many people in your life would you consider close friends or companions you trust?	0.07662835249042150
