# Supplementary material for: Use of generative AI for health among urban youth in Pakistan: A mixed-methods study
Source: PLOS Digit Health. 2026 Apr 6;5(4):e0001353. doi: 10.1371/journal.pdig.0001353 (PMC13052884; doi:10.1371/journal.pdig.0001353)
Supplement: Attachment — Submitted filename: Response to Reviewers.docx [file pdig.0001353.s011.docx]

Dear Editors & Staff,

Thank you very much for your decision letter and for the thoughtful, detailed feedback from the reviewers. We are grateful for the opportunity.

In this revised version, we have (i) strengthened the framing of the study as hypothesis-generating and exploratory early evidence from digitally connected urban youth in Pakistan, (ii) clarified our sampling, outcome definitions, and handling of missing data, (iii) expanded details on our mixed-methods integration and qualitative rigor, and (iv) addressed all journal requirements regarding ethics, author summary, data sharing, and figure licensing.

Below, we respond point-by-point to each comment from the reviewers.

Note: Supplement Figure 1 has been made via DataWrapper now. All diagrams have also been fixed in terms of dimensions via the PLOS NAAS tool.

We have added a new supplement that does all of the extra quantitative analyses requested. We have also included another figure that visually maps our results into a conceptual model, in line with reviewer comments on returning back to socio-ecological theory to understand GAI use.

All my best,
Ahsan & team

**Comment 1**

Framing and novelty

The manuscript convincingly positions itself within early LMIC evidence on GAI in health, but claims of being “first” should be softened to “among the first.” Limit claims to digitally connected urban youth to reflect the actual sampling frame.

**Response 1**

Thank you, novelty claims have been tempered we use ‘early evidence’ and ‘digitally connected urban youth’ throughout the manuscript: for example, “one of the first large-sample LMIC estimates” now reads “among some of the first large-sample LMIC” (discussion section).

**Comment 2**

Sampling and bias: Recruitment through social-media and campus networks may over-represent tech-savvy respondents. The manuscript should describe duplicate-entry safeguards (timestamps, de-duplication by contact info, CAPTCHA, etc.) and provide city/region distribution and recruitment-to-completion rates. If feasible, include a sensitivity check or demographic weighting showing whether the 69 % prevalence remains stable.

**Response 2**

Thank you for raising these important points.

We have now revised the limitations section to explicitly note how our methods may be: “introducing potential sampling bias and restricting generalizability to rural Pakistan; an English-literate, tech-savvy, digitally connected, largely urban sample that likely overestimates GAI exposure and use”

We did not collect any data on IPs or direct identifiers. This was an ethical choice made deliberately, with the understanding the duplication detection will not be possible. However, we maintained strong M&E throughout the process, as stated in the manuscript. Further, incentive to duplicate entries is low, given the minimal incentive. For context: 5000 PKR is 13 pounds. Finally and most importantly: Google forms already has a robust system, one email = one response, creating an entire new email address is a barrier to duplication.

We did not collect data on cities/geographies given the exploratory nature within an urban DC sample, however, we note that the sample, owing to our university, NGO, and social media partners, overrepresents Karachi, Lahore, Islamabad. As such, it is not possible to do demographic weighting by city/province, etc.

**Comment 3**

Outcome definition Clarify exactly how “use of generative AI for health” was measured: include the wording of survey items, response options, and the recall period (e.g., “ever,” “past 12 months”). The precise definition is crucial for interpreting the reported 69 % figure and topic-specific patterns.

**Response 3**

Thank you for your comment. A full list of exact survey items with the exact words used is now available in the Supplement. In this context, please see section 2 question 1:

Have you ever used a generative AI platform to ask about a health-related concern?

(In the manuscript: Methods section, Quantitative Survey, paragraph 2)

**Comment 4**

Model specification The multivariable logistic regression is appropriate but needs fuller reporting:

• Describe the scale and linearity assumption for ordinal predictors such as “trust in AI.” If necessary, test non-linearity or use categorical coding.
• Add AUC, calibration plot, and Brier score alongside McFadden’s R².
• Consider key theory-driven interactions (e.g., gender × condition, trust × confidence).
• Report the exact number of parameters and events-per-parameter ratio.
• The ICC confidence interval extending beyond 1.0 must be corrected.

**Response 4**

Thank you for your excellent comment - these have been addressed by adding a new supplement.

The supplement addresses the following:

1. Includes a comparison with categorical coding
2. AUC, Brier score, and the calibration plot
3. Both such theory-based interactions have been explored in the Supplement

Re. ICC: Our primary analysis uses a single-level multivariable logistic regression model without random effects, and we do not estimate or report an ICC or its confidence interval in the manuscript or supplement. We have re-checked all text and tables to confirm that no ICC values appear and therefore no correction is required. Please let us know if you meant something else.

**Comment 5**

Missing data Listwise deletion for ≤3 % missingness is acceptable, but summarize missingness by variable and, if possible, show that results are robust to simple imputation.

**Response 5**

Thank you for your comment, the added Supplement now also shows a totally summary of missingness by variable. As expected, this is highest for the question inquiring about users’ sexual orientation (LMIC Muslim context) i.e., our formative research matches this expectation.

**Comment 6**

Qualitative rigor The interviews focus on self-identified routine users, which may bias themes toward pro-use narratives. Explicitly acknowledge this and note any dissenting or negative cases. Include a brief participant-characteristics table (age, gender, city, prior digital-health experience).

**Response 6**

Thank. We did include age, gender, city and other demographic information, but had to remove it, as any more than 3 identifiers might make the participants easily identifiable, especially as they discuss issues relating to personal struggles, sexual orientation, HIV, AIDs, etc.

**Comment 7**

Integration clarity The joint-display integration is effective. Strengthen it with one concrete example of how a quantitative finding guided qualitative questioning or vice versa, to demonstrate procedural—not just interpretive—mixing.

**Response 7**

Thank you for your excellent comment, this has been addressed in two ways: first, we have included a visual conceptual model (Figure 5) which visualises the joint display better.

Furthermore: section 2.5’s final sentence includes a procedural example:

“e.g., when early survey analyses showing higher GAI use among women and youth with mental or physical conditions led, we adapted the interview guide to probe more directly around gendered stigma, emotional burden, and how these young people decide when to use GAI, seek clinicians, (or both). Furthermore, throughout data collection, we used joint displays and narrative weaving during weekly team presentations to assess convergence, divergence, and other signals”

**Comment 8**

Language and causal tone Replace causal verbs with associative phrasing (“linked to,” “associated with”). In the implications section, offer concrete harm-reduction practices for youth (e.g., verifying AI health advice, seeking clinician confirmation) and touch on governance needs for local platforms.

**Response 8**

Thank you for seeking clarification.

We have scanned the manuscript to ensure no causal language is used, e.g., the quantitative findings consistently use associative, non-causal phrasing aligned with logistic regression (e.g., “higher odds,” “associated with,” “more likely among,” “strongly predicted use”).

The discussion also outlines concrete harm-reduction practices: youth demonstrate awareness of GAI limitations, routinely verify AI advice with clinicians, triangulate responses with diagnoses and prescriptions, and use GAI to organize symptoms and prepare for medical visits. These practices are formalized in the implications/policy section through recommendations to treat AI literacy as health literacy and to teach critical appraisal skills.

Finally, governance needs for local platforms are embedded in the policy implications, which emphasize equity-first guardrails, cultural and linguistic inclusion, transparency, integration into national digital health plans, and youth co-design.

**Comment 9**

Ethics and openness Ethics approval and OSF sharing are properly noted. Place the OSF link prominently in the Data-Availability statement and include a short description of what is hosted there (dataset, survey, codebook).

**Response 9**

We have revised the Data Availability statement to make the OSF link more prominent and to clarify what is shared. The statement now notes that the OSF repository includes the de-identified dataset, the full survey and codebook, all R scripts used for cleaning and analysis, and process-mapping figures. The link has also been updated from OSF link to a DOI for ease.

**Comment 10**

Minor comments

Specify exact anchors for the “trust” and “confidence” scales and note whether they were adapted from validated tools. In regression tables, clearly mark reference groups and show non-missing n per variable.

Provide a simple participant-flow diagram (views → starts → completes → analyzed). Label multiple χ² comparisons as exploratory or apply false-discovery-rate control.

referring to these studies will enhance your argument
<https://doi.org/10.1186/s12909-024-05406-1>
<https://doi.org/10.1007/s00261-025-04810-5>

Minor proofreading: eliminate repeated phrases and ensure consistent tense

**Response 10**

Thank you for the comment.

Anchors have been specified. Further: as noted in the Methods - this survey was developed via formative research, and meant to be mobile optimized, to maximize N, and minimize drop off. The regression table already shows reference groups. Missingness is reported in the Appendix.

Our data collection tool (Google forms) does not collect data on views > starts > completes, etc. But we will consider coming up with creative ways to monitor this for our future projects.

Repeated phrases have been eliminated and tense consistency has been re-checked.

The study about attitudes towards AI and its implications for uptake has been added in the introduction. The other has to do with radiology so we are unsure where it fits.

**Comment 11**

Introduction

The section in which the study objectives are presented in the Introduction (i.e., “Accordingly, this paper contributes”) would be better positioned at the end of the Introduction to improve the logical flow and clarity of the manuscript. It is recommended that the authors describe more studies from low- and middle-income countries (LMICs) in the Introduction to provide a broader contextual background and better situate their research within the existing literature.

**Response 11**

Thank you for this helpful comment.

We have (1) moved the “Accordingly, this paper contributes…” paragraph to the end of the Introduction so that the objectives now follow the full contextualisation of GAI, youth health, and LMIC gaps, and (2) expanded the LMIC-focused background e.g., by citing studies like Wani and colleagues (2024) and a new study by Mina and colleagues (published last week).

**Comment 12**

Methods

In “Quantitative Survey”: Providing additional details on the handling of missing data

Draw Figure 1 in a larger, bold font to make it more readable.

In “Qualitative Interviews”: Consider providing additional clarification on the participant selection criteria and the rationale for the qualitative sample size (n = 20).

A brief description of how qualitative and quantitative findings were integrated in practice would help demonstrate the robustness of the convergent mixed-methods approach.

**Response 12**

Thank you for these helpful suggestions.

In the Quantitative Survey section, we state that missing data were minimal (≤3% per variable), handled via listwise deletion, and detailed in a new Supplement.

Figure 1 has been reformatted with larger, bolder text for readability. We will also check this during the proofing process. In the Qualitative Interviews section, we clarify that participants were recruited via social media as routine GAI-for-health users and that the sample size (n = 20) was pre-specified and justified using the concept of information power. We also briefly describe in the Convergent Design subsection how quantitative and qualitative findings were integrated (concurrent collection, iterative cross-talk, and joint displays); there also now a chart on OSF on the integration of the two strands.

The inclusion of another figure (Figure 5) also better visually communicates the synthesis of these findings while also directly (and more effectively) answering the RQ.

**Comment 13**

Result

The results should more explicitly connect with the study’s stated objectives, particularly regarding which socio-ecological levels (individual, interpersonal, community, or system) the findings correspond to.

Both significant and non-significant associations should be reported systematically to avoid selective emphasis and to give a balanced view of the results.

**Response 13**

Thank you for your excellent suggestion. We return to the socio-eco model in the discussion section, via Figure 5. Which is then continually referenced throughout the discussion (either directly e.g., “see Figure 5” or by mirroring its language).

Also: we have sign-posted this better, under the Results (but before 3.1).

We report both significant and non-significant associations.

**Comment 14**

The discussion effectively interprets findings through the socio-ecological framework, but the link between individual-, community-, and system-level determinants could be made more explicit.

Consider adding a short integrative paragraph synthesizing how these levels interact in shaping youth GAI use.

The contrast with HIC data is informative; however, adding a sentence on why structural and cultural differences (e.g., stigma, infrastructure, digital literacy) may explain higher uptake in Pakistan would deepen the analysis.

The conclusion is concise and well articulated. You might consider ending with a more forward-looking statement—e.g., how GAI could be ethically integrated into LMIC health systems or monitored within national digital health agendas.

**Response 14**

Thank you for your helpful comment. We have added Figure 5, which 1) speaks directly to the layered nature of our inquiry, and 2) directly shows how this shapes GAI use.

A paragraph under the results now makes this more explicit, and guides the reader better throughout the narrative, ultimately leading to the visual display.

**Comment 15**

The study’s methodological transparency and ethical conduct are highly commendable. However work the following: Expand the description of instruments and analytic frameworks within the main text. Clarify sampling strategy limitations and justify analytical decisions. Ensure that all analytic scripts and supporting documentation are included in OSF, with explicit links/references. Add detail about data sharing and privacy for both survey and interview components. The implications and conclusion are clear. Highlight future directions for addressing sampling and measurement gaps, and potentially new methodologies for reaching less-connected youth populations as digital access expands.

**Response 15**

Thank you for your kind words.

A fuller description of each instrument is available in the Supplements.

Sampling strategy limitations are now 1) more explicitly noted, and 2) more often (see strengths and limitations, and discussion section). OSF now includes process flow that maps decisions.

All R scripts and additional documents are now in OSF.

Discussion and conclusion now highlight future directions, and focus on AI-literacy considerations (equity in use), and we call on other researchers to use better proxies for SES.
