# Supplementary material for: Use of generative AI for health among urban youth in Pakistan: A mixed-methods study
Source: PLOS Digit Health. 2026 Apr 6;5(4):e0001353. doi: 10.1371/journal.pdig.0001353 (PMC13052884; doi:10.1371/journal.pdig.0001353)
Supplement: Attachment — Submitted filename: Response_to_Reviewers_auresp_2.docx [file pdig.0001353.s012.docx]

**Response to Reviewers**

**Reviewer Comment:**

you should strengthen the discussion by explicitly addressing: How digital literacy, language, and connectivity may shape both access and health-seeking behavior. The potential for systematic overestimation of GAI adoption. The role of algorithmic exposure and platform familiarity in driving reported use. Whether this population represents early adopters or structurally privileged subgroups. The discussion would benefit from framing this cohort as a “leading edge” or innovation adoption group rather than a representative youth population.

There is something fascinating hiding inside this paper that deserves to be pushed even further. It is not just about AI use. It is about how health systems fail and how young people quietly build shadow infrastructures of care when institutions do not meet their needs. That angle, if sharpened, could turn this from a descriptive study into a foundational piece in precision public health and digital resilience research.

**Authors’ Response:**

Thank you for your very kind words and the excellent comments!

We have substantially revised the discussion section to explicitly focus on structural privilege. We draw more heavily on the works of Shim and colleagues (health cultural capital) to sharpen this angle. Also, we have made similar changes to the joint table.
